# Helicopter parenting and college student depression: the mediating effect of physical self-esteem

**DOI:** 10.3389/fpsyt.2023.1329248

**Published:** 2024-01-09

**Authors:** Chaolian Wang, Heng Shi, Geng Li

**Affiliations:** ^1^Center for Textbook Compilation and Translation of Xizang Autonomous Region, Lhasa, China; ^2^Center for Disease Control and Prevention of Xizang Autonomous Region, Lhasa, China; ^3^College of Physical Education, Hunan Normal University, Changsha, China

**Keywords:** helicopter parenting, depression, college students, physical self-esteem, mediating effect

## Abstract

**Background:**

Depression is one of the most common and prevalent mental disorders, and college students are a high-risk group for depression. Helicopter parenting plays an important role in depression, but the mechanism is still ambiguous. Therefore, this study investigates the specific impact and mechanism of helicopter parenting on college students’ depression.

**Methods:**

Employing a questionnaire-based approach, we assessed the relationship between helicopter parenting, Physical self-esteem, and depression. The questionnaire comprised three scales: the Helicopter Parenting Scale, Physical Self-Esteem Scale, and Self-Rating Depression Scale. The study sample included 539 university (average age 18.84 ± 1.1 years; 184 males and 355 females).

**Results:**

Helicopter parenting demonstrated significant negative predict with physical self-esteem (*β* = −0.75, *p* < 0.001), and positive predict depression (*β* = 0.33, *p* < 0.001). Helicopter parenting impacts depression among college students through two channels: solely via physical self-esteem (mediating effect value: 0.66), and through direct influence (effect value: 0.64).

**Conclusion:**

The insights from this study address the two pivotal questions about “why” and “how” helicopter parenting influences depression in college students, offering recommendations for managing depressive moods among college students.

## 1 Introduction

The concept of “helicopter parenting,” which originated in Western societies, refers to a style of over-involved parenting, where parents excessively intervene in the lives of their young adult children (1). This style is often marked by heightened behavioral control and undue restrictions on children’s autonomy (2). Although helicopter parenting aims to promote children’s success, it may paradoxically increase the risk to their psychological health (3–5). This trend of parenting is becoming increasingly common in China (2, 6–8), where the one-child policy, a fundamental element of China’s traditional family structure, has intensified parental scrutiny and expectations on the only child, leading to a unique cultural adaptation of helicopter parenting (9, 10). The growing phenomenon of helicopter parenting in China calls for an in-depth exploration of its effects, especially considering its potential to initiate or worsen mental health issues.

Amidst the mounting concern for psychological well-being, depression emerges as a critical issue closely entwined with the parenting styles prevalent in society (11–13). College students, already navigating the tumultuous transition from adolescence to adulthood, confront additional stressors that may be exacerbated by overbearing parental involvement. This is evidenced by the alarming statistics revealing that 24.71% of Chinese college students exhibit depressive symptoms. This figure mirrors the increasing prevalence of over-parenting and is continuing to rise (14). These stressors, ranging from academic pressure to the challenges of environmental adaptation and future planning, place college students at heightened risk for depression (15, 16), a condition that not only impairs cognitive functions and executive capacity but also predisposes them to social withdrawal and suicidality (17). Therefore, it becomes especially urgent and important to deeply study the mechanisms between helicopter parenting and college students’ depression.

### 1.1 Helicopter parenting and college students’ depression

According to the Social-Ecological Systems Theory, individuals are situated within specific environmental systems, and individual psychological development is the result of continuous interaction with the social environmental system (18). Among these environmental systems, the family environment, is the earliest growth setting for individuals (19). Further, the parenting style within the family environment is one of the most direct and closely related influencing factors during an individual’s growth process, having a long-term and progressive impact on psychological health (14). Existing research indicates that depression is not innate, and parenting style plays a significant role in influencing depression (20). Specifically, parents who encourage autonomy allow their children to explore interests and minimize control and pressure to the greatest extent, which helps inhibit the development of depressive feelings (21). However, if parents adopt controlling methods (e.g., decision interference, deprivation, and supervision) to guide their children’s behavior, these children will experience reduced autonomy, leading to an accumulation of depressive emotions (22). Based on this, the influence of parenting styles on college students’ depression should not be underestimated.

Recent studies have underlined the link between helicopter parenting and depression in young adults, with heightened parental involvement potentially leading to feelings of isolation among this demographic (20). This isolation may, in turn, influence young adults to attribute their life outcomes to external factors, such as luck or opportunity, rather than their efforts and abilities (23). Such external attribution is known to exacerbate depressive feelings when facing life’s challenges (24). Supporting this, research has consistently found a significant relationship between helicopter parenting and the emergence of neurotic traits that are closely associated with depression (25, 26). Additionally, there is a body of evidence indicating that individuals raised by helicopter parents have a higher propensity to use antidepressant medications (7). Based on these insights, this study hypothesizes that helicopter parenting will predict depression in college students (Hypothesis 1).

### 1.2 The mediating role of physical self-esteem

Self-determination theory posits that the fulfillment of basic psychological needs for autonomy, competence, and relatedness is vital for an individual’s well-being (27). Helicopter parenting, characterized by intrusive and controlling behaviors, can undermine these needs by impeding students’ autonomy and hampering the development of competence. This encroachment may lead to diminished physical self-esteem, as students’ confidence in their body image and physical capabilities—an intrinsic aspect of the need for competence—becomes compromised (28). Furthermore, low physical self-esteem can foster a negative self-concept, a well-established precursor to depressive symptoms (29). Given these insights, it invites consideration that physical self-esteem might play a mediating role in how helicopter parenting affects depression in college students, suggesting a nuanced pathway that warrants further exploration.

Building on this theoretical foundation, physical self-esteem stands as a core component of overall self-esteem and begins to take shape early in one’s self-awareness development. It reflects an individual’s self-perception and valuation of their appearance and physical abilities (30). Evidence suggests that physical self-esteem plays a significant role in regulating depression levels, with those possessing higher physical self-esteem more likely to utilize support networks effectively in the face of adversity (31, 32). These support mechanisms can help alleviate stress and enhance adaptability, potentially curbing the development of depressive symptoms (33). Conversely, low physical self-esteem may lead to a decline in coping effectiveness, increasing susceptibility to mental distress and the accumulation of depressive feelings. As such, this study posits that physical self-esteem may predict the presence of depression in college students (Hypothesis 2).

While prior research has focused on the relationship between helicopter parenting and general self-esteem (22), the specific impact of such parenting on physical self-worth remains underexplored. The Social Comparison Theory complements the Self-Determination Theory by suggesting that individuals form their self-perceptions by comparing themselves to their peers (34). Positive parenting styles that foster autonomy can enhance physical self-esteem as individuals affirm their control in these comparisons (35). However, helicopter parenting, by restricting autonomy and emphasizing success, may negatively influence this process. The detrimental effects of helicopter parenting on autonomy and subsequent physical self-worth become even more pronounced when viewed through the lens of comparative self-evaluation, potentially diminishing an individual’s physical self-esteem (36). In light of these considerations, this study hypothesizes that physical self-esteem will function as a mediating variable, linking helicopter parenting with college students’ depression (Hypothesis 3).

**Figure 1:**
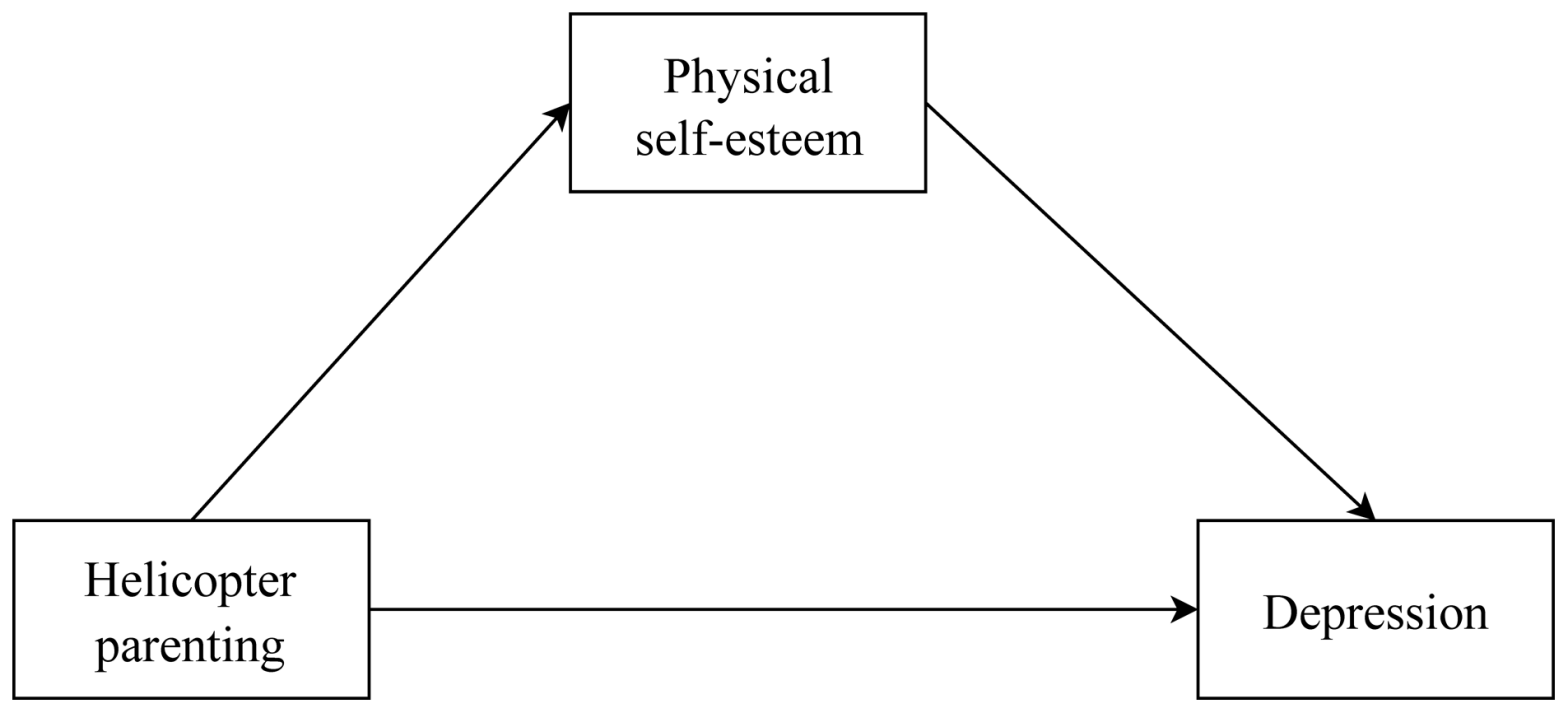
Mediation hypothesis model.

In summary, this study endeavors to articulate a comprehensive mediation model (Figure 1) that disentangles the nuanced mechanisms between helicopter parenting and depression in college students, with physical self-esteem serving as a pivotal mediating factor.

## 2 Research methods

### 2.1 Participants

Utilizing convenience cluster sampling, this study selected 600 undergraduates from 20 classes across four universities in China. The data collection was conducted in May 2023. Participants were required to complete offline paper questionnaires. Before the survey, an “Informed Consent Form for Survey” was distributed, ensuring that consent was obtained from participants who were informed about and agreed to participate in the study. Standardized instructions were provided on-site, detailing necessary precautions and emphasizing the principles of voluntary participation and the confidentiality of the survey responses.

Under the supervision of research assistants, participants filled out the paper questionnaires based on their true conditions, ensuring the integrity and accuracy of the data collection process. Each student completed three questionnaires: the “Helicopter Parenting Scale,” the “Physical Self-Esteem Scale,” and the “Depression Self-Assessment Scale.” Out of the 594 individuals who agreed to participate and completed the survey, 55 invalid questionnaires were excluded (11 blank, 20 with missing answers, and 24 with patterned answers), resulting in 539 valid questionnaires. The demographic composition of the valid responses included an average age of 18.84 ± 1.1 years, comprising 184 males and 355 females. The effective response rate was 90.74%. The questionnaire completion process took approximately 15 min for each participant.

### 2.2 Measures

#### 2.2.1 Helicopter parenting

The helicopter parenting scale, originally developed by Padilla-Walker et al. (1) and later revised by Lin (6), was employed to gage the extent of helicopter parenting experienced by students in China. The scale consists of 5 test items, for example, “My parents make crucial decisions for me, like where I should live, work, or the courses I should enroll in.” The scale uses a 5-point scoring system, with 1 being “completely disagree” and 5 being “completely agree.” A higher score indicates a higher level of helicopter parenting experienced by the college student. In the original validation study by Padilla-Walker et al. (1), the Cronbach’s α coefficient was reported as 0.77, and the Cronbach’s α coefficient in this study is 0.715, indicating a consistent reliability across different contexts.

#### 2.2.2 Physical self-esteem

The Physical Self-Perception Profile (PSPP), originally developed by Fox and Corbin (37) and later revised by Xu and Yao (38), was employed to measure the physical self-esteem of students in China. The scale consists of 30 test items, encompassing five dimensions: athletic competence, physical condition, body condition, self-worth, and physical attractiveness. For example, “I think it’s easy for me to keep my body attractive.” The scale uses a 4-point scoring system, with 1 being “strongly disagree” and 4 being “strongly agree.” A higher score indicates a higher level of physical self-esteem among college students. In the original validation study by Fox and Corbin (37), the Cronbach’s α coefficient was reported as 0.92, and the Cronbach’s α coefficient of this study is 0.967, indicating a consistent reliability across different contexts.

#### 2.2.3 Depression

The Self-Rating Depression Scale (SDS) was originally developed by Zung (39) and later revised by Yuan et al. (40) to assess depression in China. The scale consists of 20 test items, covering four dimensions: psychological-affective symptoms, somatic disturbances, psychomotor disturbances, and psychological impediments of depression. For example, “I feel useful and indispensable.” The scale uses a 4-point scoring system, with 1 being “strongly disagree” and 4 being “strongly agree.” A higher score indicates a higher frequency of depressive symptoms in the college student. In the original validation study by Zung (39), the Cronbach’s α coefficient was reported as 0.79 (41), and the Cronbach’s α coefficient of this study is 0.794, indicating a consistent reliability across different contexts.

#### 2.2.4 Personal demographics

Research suggests that personal demographics such as gender, age, and family socioeconomic status can significantly impact parenting styles and depression (42, 43). Building upon this foundation, the present study incorporates findings from Qu (44) and Ge (45) concerning Chinese college students. Essential personal information—like gender, age, annual household income, and the educational levels of both parents, as well as perceived family social class—was gathered to control potential confounding variables. A higher score relating to annual household income, parents’ education levels, and perceived family social class denotes a higher standing in those respective domains.

### 2.3 Statistical analysis

In this study, we conducted statistical analyses using SPSS 25 for Windows 10. The analysis accounted for missing data, which were minimal and did not significantly impact the overall dataset. The missing values were imputed using multiple imputation techniques. Sum scores for all measures were computed to investigate the research questions and to assess the hypothesized relationships among variables. Initially, a Harman single-factor test, following Zhou and Long's (46) guidelines, was applied to address and evaluate potential common method bias. This was succeeded by an examination of the inter-variable relationships using Pearson correlation coefficients, which laid the groundwork for understanding the dynamics between helicopter parenting, physical self-esteem, and depression.

We then progressed to regression analysis, employing Model 4 of PROCESS macro, to discern the direct effects and to scrutinize both simple and mediated relationships present in the data (13). To rigorously evaluate the mediation effects, we used the bias-corrected non-parametric percentile Bootstrap method. This sophisticated approach allowed for the creation of a sampling distribution of indirect effects, leading to the construction of confidence intervals for the mediation effect size (47). Each stage of analysis was carefully chosen and conducted with the aim of thoroughly examining the data and precisely validating the theoretical framework proposed by this research.

## 3 Results

### 3.1 Common method bias test

In the present study, measures were taken to address potential common method bias by employing anonymous surveys and incorporating reverse coding for particular items, as suggested by Zhou and Long (46). To further evaluate the presence of common method bias, a Harman single-factor analysis was conducted using SPSS 25.0. The analysis revealed eleven factors with eigenvalues surpassing 1. Notably, the principal factor accounted for only 31.65% of the variance, falling short of the critical 40% threshold. Thus, it can be concluded that this research was not influenced by common method bias.

### 3.2 The correlation between the study variables

This study utilized SPSS 25.0’s Pearson correlation test to compute the mean, standard deviation, and correlations of various variables. As the main results are shown in Table 1, the correlation of helicopter parenting was negatively related to physical self-esteem (*r* = −0.76, *p* < 0.001) and gender (*r* = −0.12, *p* < 0.001), positively related to depression (*r* = 0.68, *p* = 0.012). The correlation of physical self-esteem was negatively related to physical self-esteem (*r* = −0.76, *p* < 0.001), depression (*r* = −0.71, *p* < 0.001), and mother’s education level (*r* = −0.09, *p* = 0.032). The correlation of depression was negatively related to physical self-esteem (*r* = −0.71, *p* < 0.001), and positively related to helicopter parenting (*r* = 0.68, *p* < 0.001). However, the correlation of family socioeconomic status was not related to helicopter parenting and depression (*p* > 0.05), but gender was negatively related to helicopter parenting (*r* = −0.12, *p* = 0.018).

**Table 1:** Descriptive statistics and interrelations among all observed variables.

Variables	M	SD	1	2	3	4	5	6	7	8	9
1. Helicopter parenting	12.85	7.1	1								
2. Physical self-esteem	76.97	6.03	−0.76***	1							
3. Depression	55.41	12.38	0.68***	−0.71***	1						
4. Gender	0.34	0.47	−0.12**	0.12**	−0.07	1					
5. Age	18.84	1.10	0.00	0.02	0.00	0.07	1				
6. Annual household income	4.31	1.53	−0.04	0.05	−0.06	0.04	0.08	1			
7. Father’s education level	2.87	1.16	0.04	−0.02	0.00	−0.03	0.07	0.27***	1		
8. Mother’s education level	2.65	1.21	0.06	−0.09*	0.04	−0.09*	0.01	0.24***	0.61***	1	
9. Subjective family social class	2.40	0.77	−0.07	0.07	−0.07	−0.04	−0.01	0.30***	0.36***	0.35***	1

^***^p < 0.001; ^**^p < 0.005; ^*^p < 0.05.

### 3.3 The regression analysis

Based on the mediation testing method by Wen and Ye (2014), this research used Model 4 in SPSS plugin PROCESS (13), with helicopter parenting as the independent variable, depression as the dependent variable, physical self-esteem as the mediating variable, and gender, age, annual household income, father’s education level, mother’s education level, and subjective family social class were taken as control variables. The results of the regression analysis are shown in Table 2. Specifically, Model 1 was constructed to examine the impact of helicopter parenting on physical self-esteem. The results indicated that helicopter parenting negatively predicted physical self-esteem (*β* = −0.75, *p* < 0.001). This suggests that higher levels of helicopter parenting are associated with lower levels of physical self-esteem among college students. However, Model 1 accounted for 56% of the variance in physical self-esteem, with an F-statistic of 84.95, denoting that the model was a good fit for the data.

**Table 2:** The mediation model from helicopter parenting to depression.

	Model 1			Model 2		
	Physical self-esteem			Depression		
	*β*	SE	*t*	*β*	SE	*t*
Helicopter parenting	−0.75	0.14	9.46^***^	0.33	0.08	7.51^***^
Body self-esteem				−0.46	0.01	−10.38^***^
*R* ^2^	0.56			0.58		
*F*	84.95			108.14		

^***^p < 0.001.

**Figure 2:**
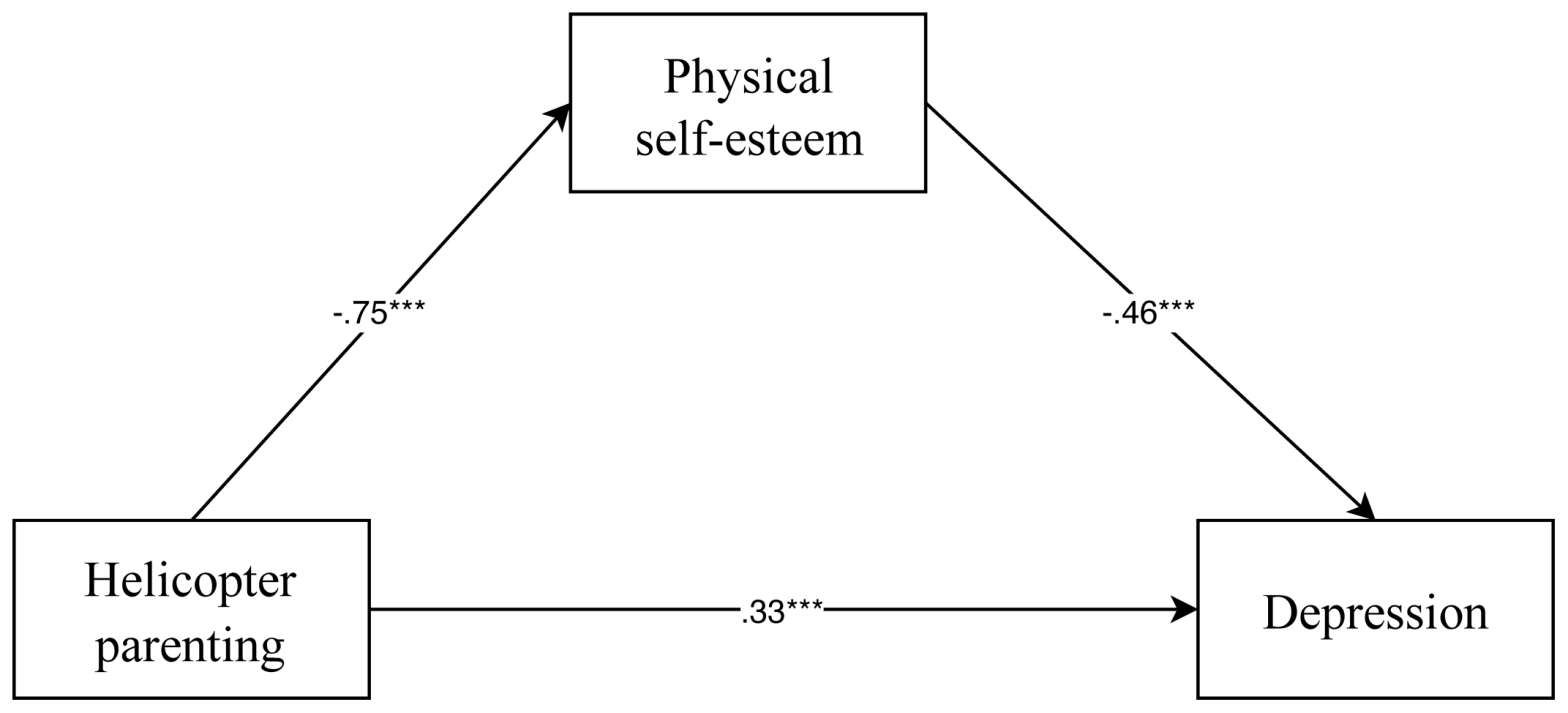
Model of the mediated role of physical self-esteem in the relationship between helicopter parenting and depression.

In Model 2, the direct relationship between physical self-esteem and depression was examined alongside the role of helicopter parenting. Here, physical self-esteem negatively predicted depression (*β* = −0.46, *p* < 0.001), indicating that higher physical self-esteem is associated with lower depression scores. Additionally, helicopter parenting positively predicted depression (*β* = 0.33, *p* < 0.001), suggesting that more helicopter parenting is correlated with higher levels of depression, supporting hypothesis 2. However, Model 2 explained 58% of the variance in depression, with an F-statistic of 108.14, confirming the model’s strong explanatory power. Concurrently, it was found that all the standardized path coefficients in the model were significant (Figure 2).

### 3.4 The mediating effect analysis

This study applied the bias-corrected non-parametric percentile Bootstrap method, entailing 5,000 resamples, to rigorously assess the mediation effects. All tests of statistical significance were conducted with an alpha level set at 0.05. The results, detailed in Table 3, include 95% confidence intervals for the indirect effects, offering a precise estimation of the effect sizes and affirming the reliability of the mediation analysis. Specifically, a significant indirect effect of helicopter parenting on depression via physical self-esteem, evidenced by an effect size of 0.66 and a 95% CI [0.49, 0.84], which accounted for 50.15% of the total effect. The direct effect of helicopter parenting on depression was also notable at 0.64 (95% CI [0.47, 0.80]), contributing 48.85% to the total effect. The non-inclusion of zero in the 95% confidence intervals across all pathways confirms the significant mediation effect, thereby supporting Hypothesis 3.

**Table 3:** Bootstrap mediating effects of helicopter parenting and depression.

Paths	Effect	BootSE	95%CI		Relative mediating effect
Total effect	1.31	0.06	1.19	1.43	100%
Direct effect	0.64	0.08	0.47	0.80	48.85%
Indirect effect	0.66	0.08	0.49	0.84	50.15%

Total effect: Direct effect + Indirect effect; Direct effect: Helicopter parenting → Depression; Indirect effect: Helicopter parenting → Physical self-esteem → Depression.

## 4 Discussion

In this study, we constructed a structural model encompassing helicopter parenting, physical self-esteem, and depression. The model posits that while helicopter parenting has a direct impact on depression, physical self-esteem mediates the relationship between helicopter parenting and depression. The findings corroborated Hypotheses 1–3, enriching our comprehension of how helicopter parenting affects college students’ depressive symptoms. Such insights offer empirical grounding for future interventions.

### 4.1 Helicopter parenting and depression

The results of this study indicate that helicopter parenting positively predicts depression, corroborating hypothesis 1. This observation is consistent with prior research (48, 49), bolstering the tenets of the social-ecological theory. Within the family system, parenting styles play a pivotal role in shaping an individual’s psychological well-being (35, 36). Past studies have also highlighted achievement anxiety and diminished autonomy as notable precursors to depression (50). Given that helicopter parenting is typified by undue protection and control, it stifles individual autonomy and a sense of achievement (51). Consequently, helicopter parenting stands out as a salient predictor of depression.

Notably, when comparing the correlation coefficients between helicopter parenting and depression among college students in previous research, this study exhibits certain disparities. Schiffrin et al. (26) reported a value of 0.27, Turner et al. (52) found 0.2, and Cook (53) documented 0.12. In contrast, this study yielded a higher coefficient of 0.68. Such variation can likely be attributed to distinct cultural and national contexts. Although China has loosened its one-child policy, its enduring legacy means many families have a single child. Even when attending college away from home, students may still experience helicopter parenting, perhaps through distant communication (54). Additionally, rooted Confucian values emphasize filial piety and reverence for elders, potentially leading parents to express heightened concern about their children’s growth and academics (55). This concern may evolve into the overprotection synonymous with helicopter parenting. Conversely, in Western cultures, the emphasis on individual autonomy may prompt parents to grant their children more freedom, minimizing their controlling tendencies. By delving into the context of Chinese education, this study broadens the applicability of the social-ecological framework. Moreover, the results underscore the potential merit of refining parenting techniques to prevent the onset of depressive symptoms in young adults.

### 4.2 The mediating role of physical self-esteem

This study substantiated the predictive nature of physical self-esteem on depression among college students, while also affirming its mediating role between helicopter parenting and such depression. Both Hypotheses 2 and 3 were supported. This aligns, in part, with prior empirical findings (28), corroborating the social comparison theory (34). Historically, physical self-esteem has been recognized as a significant predictor of depression (56). Furthermore, in a 16-week study by He and Ji (28), a causative link was identified between individual physical self-esteem and their depression levels. This research further delves into the precursors of physical self-esteem fluctuations. Specifically, individuals tend to assess their capacities and accomplishments through comparison with others. The nature of helicopter parenting may inadvertently compel children to engage excessively in such comparisons, overshadowing their individual growth (57). Consequently, this predisposes them to negative self-assessments regarding physical competencies and appearance, undermining their physical self-worth. Moreover, influenced by helicopter parenting, students with diminished physical self-esteem may gravitate towards unproductive coping mechanisms like self-devaluation and avoidance when confronted with stressors, culminating in depressive sentiments (24).

Within the sphere of helicopter parenting, parents’ pronounced control often infringes on an individual’s autonomy, channeling much of their focus into academics and comparable endeavors. This not only directly heightens college students’ susceptibility to depression but also inadvertently diminishes their physical self-esteem during self-comparisons (50, 58), further exacerbating depression. In summation, this study’s mediation model provides an elucidated understanding of the intricate processes by which helicopter parenting modulates college students’ depression levels.

### 4.3 Limitations and future direction

This research utilized a cross-sectional study design, which facilitated the identification of relationships among the variables under examination, thereby laying the groundwork for future empirical investigations. Nonetheless, to ascertain causality among these variables, embarking on a longitudinal experimental approach would be indispensable. Additionally, our data collection hinged predominantly on self-reported measures. Although this method provided a foundational perspective, it is paramount for subsequent studies to employ more objective means. While the crux of this study was the exploration of physical self-esteem as a mediator between helicopter parenting and depression in college students, it is pertinent to acknowledge the potential significance of other mediatory factors. Variables such as self-efficacy, self-awareness, and overall physical health could be pivotal in this dynamic, thereby heralding fresh avenues for future inquiries.

## 5 Conclusion

Upon surveying 539 college students, our research demystified the intricate relationship between helicopter parenting, physical self-esteem, and depression. We observed a pronounced positive relationship between helicopter parenting and depression and a discernible negative tie with physical self-esteem. Moreover, helicopter parenting did not show any substantial association with family socio-economic status. Our findings suggest that helicopter parenting could indeed forecast variations in physical self-esteem and depression levels. Crucially, physical self-esteem stood out as a mediating factor, elucidating the connection between helicopter parenting and depression in college students. This study not only deciphers the underpinnings of how and why helicopter parenting impacts depression in college students but also propounds actionable guidelines for interventions aiming to ameliorate depressive states in this demographic.

## Data availability statement

The raw data supporting the conclusions of this article will be made available by the authors, without undue reservation.

## Ethics statement

The studies involving human participants were reviewed and approved by the Ethics Committee of the Hunan Normal University. The patients/participants provided their written informed consent to participate in this study.

## Author contributions

CW: Conceptualization, Investigation, Project administration, Supervision, Validation, Writing – original draft. HS: Conceptualization, Data curation, Investigation, Project administration, Supervision, Validation, Visualization, Writing – original draft. GL: Data curation, Formal analysis, Methodology, Resources, Software, Validation, Writing – original draft.
